# Supplementary material for: Utilization of Polymeric Micelles as a Lucrative Platform for Efficient Brain Deposition of Olanzapine as an Antischizophrenic Drug via Intranasal Delivery
Source: Pharmaceuticals (Basel). 2022 Feb 18;15(2):249. doi: 10.3390/ph15020249 (PMC8877317; doi:10.3390/ph15020249)
Supplement: Supplementary file 1 [file pharmaceuticals-15-00249-s001.zip › pharmaceuticals-1585297-supplementary.pdf]

Article

Utilization of Polymeric Micelles as a Lucrative Platform for Efficient Brain Deposition of Olanzapine as an Antischizophrenic Drug via Intranasal Delivery

Hadel A. Abo El-Enin [1,*], Marwa F. Ahmed [2], Ibrahim A. Naguib [2], Shaymaa W. El-Far [3], Mohammed M. Ghoneim [4], Izzeddin Alsalahat [5] and Hend Mohamed Abdel-Bar [6]

[1] Department of Pharmaceutics and Industrial Pharmacy,, College of Pharmacy, Taif University, P.O. Box 11099, Taif 21944, Saudi Arabia
[2] Department of Pharmaceutical Chemistry, College of Pharmacy, Taif University, P.O. Box 11099, Taif 21944, Saudi Arabia; marwa.farg@tu.edu.sa (M.F.A.); i.abdelaal@tu.edu.sa (I.A.N.).
[3] Division of Pharmaceutical Microbiology, Department of Pharmaceutics and Industrial Pharmacy, College of Pharmacy, Taif University, P.O. Box 11099, Taif 21944, Saudi Arabia; shfar@tu.edu.
[4] Department of Pharmacy Practice, College of Pharmacy, AlMaarefa University, Ad Diriyah, Riyadh 13713, Saudi Arabia; mghoneim@mcst.edu.sa
[5] UK Dementia Research Institute Cardiff, School of Medicine, Cardiff University, Cardiff CF24 1TP, UK; alsalahati@cardiff.ac.uk
[6] Department of Pharmaceutics, Faculty of Pharmacy, University of Sadat City, P.O. Box 32897, Egypt; hend.abdelbar@fop.usc.edu.eg
* Correspondence: hadel.a@tu.edu.sa (H.A.A.)

Supplementary Material

Table S1. : Model summary statistics for particle size (Y1) and for EE % (Y2).

Particle size (Y1)						
**Source**	**Std. Dev.**	**R^2**	**Adjusted R^2**	**Predicted R^2**	**PRESS**	
Linear	1.72	0.7036	0.6580	0.5359	60.42	
**Quadratic [a]**	**0.3393**	**0.9912**	**0.9867**	**0.9746**	**3.31**	**Suggested**
Special Cubic	0.3398	0.9920	0.9867	0.9758	3.15	
Cubic	0.3889	0.9930	0.9826	-6.7048	1003.07	
Special Quartic	0.3609	0.9930	0.9850	0.9720	3.65	
Quartic	0.4187	0.9933	0.9798		*	Aliased
**EE % (Y2)**						
**Source**	**Std. Dev.**	**R^2**	**Adjusted R^2**	**Predicted R^2**	**PRESS**	
Linear	3.08	0.6624	0.6105	0.4918	185.58	
**Quadratic [b]**	**0.8177**	**0.9817**	**0.9725**	**0.9489**	**18.65**	**Suggested**
Special Cubic	0.8607	0.9817	0.9696	0.9443	20.33	
Cubic	0.9593	0.9849	0.9622	-1.7837	1016.51	
Special Quartic	0.8921	0.9847	0.9673	0.9424	21.02	
Quartic	1.05	0.9850	0.9549		*	Aliased

[a] Adequate precision equals 48.49 and coefficient of variation (C.V.) % is 0.833. [b] Adequate precision equals 32.72 and coefficient of variation (C.V.) % is 1.05.

Table S2. Quantitative factor effects on the A: particle size (Y1) and B: EE % (Y2) expressed as the coefficients of the regression equations.

A: particle size (Y1)						
**Component**	**Coefficient Estimate**	**df**	**Standard Error**	**95% CI Low**	**95% CI High**	**VIF**
A-P123	49.91	1	0.7266	48.29	51.53	16.59
B-P407	42.96	1	0.7064	41.39	44.53	14.11
C-TPGS	88.44	1	5.02	77.26	99.61	192.69
AB	-30.24	1	2.42	-35.63	-24.85	17.19
AC	-68.56	1	7.48	-85.24	-51.89	81.61
BC	-80.96	1	7.29	-97.20	-64.72	74.25
**B: EE % (Y2).**						
**Component**	**Coefficient Estimate**	**df**	**Standard Error**	**95% CI Low**	**95% CI High**	**VIF**
A-P123	105.52	1	1.75	101.62	109.42	16.59
B-P407	93.43	1	1.70	89.64	97.22	14.11
C-TPGS	-5.04	1	12.09	-31.97	21.89	192.69
AB	-74.19	1	5.83	-87.17	-61.21	17.19
AC	42.05	1	18.03	1.87	82.23	81.61
BC	67.93	1	17.56	28.80	107.06	74.25

Table S3. ANOVA of the obtained data from D-Optimal design for the particle size (Y1) and EE % (Y2) of olanzapine polymeric micelles and associated p-values.

	Source	Sum of Squares	df	Mean Square	F-value	p-value	
A: particle size (Y1)	**Model**	129.04	5	25.81	224.15	< 0.0001	significant
	[1]Linear Mixture	91.60	2	45.80	397.81	< 0.0001	
	AB	18.02	1	18.02	156.48	< 0.0001	
	AC	9.67	1	9.67	83.96	< 0.0001	
	BC	14.21	1	14.21	123.41	< 0.0001	
	**Residual**	1.15	10	0.1151			
	Lack of Fit	0.2750	5	0.0550	0.3137	0.8855	not significant
	Pure Error	0.8764	5	0.1753			
	**Cor Total**	130.19	15				
	**Source**	**Sum of Squares**	**df**	**Mean Square**	**F-value**	**p-value**	
B: EE % (Y2)	**Model**	358.49	5	71.70	107.23	< 0.0001	significant
	[1]Linear Mixture	241.90	2	120.95	180.89	< 0.0001	
	AB	108.44	1	108.44	162.17	< 0.0001	
	AC	3.64	1	3.64	5.44	0.0419	
	BC	10.00	1	10.00	14.96	0.0031	
	**Residual**	6.69	10	0.6686			
	Lack of Fit	1.20	5	0.2391	0.2178	0.9401	not significant
	Pure Error	5.49	5	1.10			
	**Cor Total**	365.17	15				
